# Two birds with one stone - how to reduce the waiting list and provide effective treatment at the same time

**DOI:** 10.1186/2050-2974-2-S1-O18

**Published:** 2014-11-24

**Authors:** Katryna Henry, Jodie Sheraton

**Affiliations:** 1 Central Coast Eating Disorders Outpatient Service, Wyong, Australia

## 

Resource limitations can often result in creative solutions. This has been the case with the evolution of our parent education and support group, "Restore" which has enabled timely access to effective treatment. The Early Intervention team is a multidisciplinary team of 1.5 clinicians, (social work/ dietitian/ clinical psychologist) who offer individual, family therapy and now group parent therapy.

Treatment for eating disorders is lengthy. Our team treats up to 38 people a year with two thirds using Maudsley Family Based treatment. Until the introduction of "Restore" we had up to 13 on the waiting list at any one time. The severity, chronicity and complexity of presentations resulted in a greater demand on our services with 97% of those assessed and then recommended for early intervention treatment in 2013 being placed on the waiting list.

Our "Restore" group parent program has evolved from the Westmead Children's Hospital "Nourish" program. It incorporates elements from Multiple Family Therapy model developed by Ivan Isler and Maudsley Family Therapy, with unique nutrition education for parents and a weight/symptom monitoring component.

The Restore parent group is a 6 week educational/therapy based intervention group for parents of a child with an eating disorder and is trans diagnostic. Each cohort is evaluated using pre and post-test measures and weekly participant feedback. Outcomes to date show that "Restore" is effective in: improving access to treatment by reducing waiting times; improving progress towards the individual's weight goals; reducing Binge/Purge behaviours; increasing parental confidence to bring about the complete recovery of their child in the home setting.

The "Restore" program is a means to provide education and support to parents, provides timely, effective treatment and enables identification of those who need more intensive ongoing therapy.

This abstract was presented in the **Service Initiatives: Child and Adolescent Refeeding and FBT** stream of the 2014 ANZAED Conference.

